# Non-pharmacological treatment of psychiatric disorders in a nationwide population

**DOI:** 10.1192/j.eurpsy.2022.848

**Published:** 2022-09-01

**Authors:** S. Elkrog, M. Ernst, L. Rasmussen, R. Wesselhoeft

**Affiliations:** University of Southern Denmark, Ist - Clinical Pharmacology, Pharmacy And Environmental Medicine, Odense M, Denmark

**Keywords:** Psychotherapy, nationwide population, register, psychiatric disorders

## Abstract

**Introduction:**

Non-pharmacological treatment like psychotherapy is associated with less side effects than pharmacological treatment and is often considered first-line treatment towards psychiatric disorders. The extent and variation of psychotherapy treatment offered in Danish psychiatric clinics over time has not previously been studied.

**Objectives:**

To examine the nationwide use of psychotherapy treatment during 2001-2020 in individuals assigned with a psychiatric disorder diagnosis at Danish psychiatric clinics.

**Methods:**

All Danish individuals aged ≥ 3 years, who were registered with 1) a psychiatric disorder diagnosis (F10-F99) or 2) had a first psychotherapy treatment during the study period 1 January 2001 to 31 December 2020, were identified in the Danish National Patient Registry.

**Results:**

A total of 120,916 (27 %) study participants received psychotherapy treatment during the study period, most commonly individual psychotherapy (65 %) followed by group therapy (25 %). Adults (≥18 years) were more likely to receive therapy (34 %) than children and adolescents aged 3-17 years (15 %). The proportion of treated patients was highest among women (67 %) compared with men (33 %). The median age at first psychotherapy was 25 years (ranging from 19 to 33). 59 % of patients receiving psychotherapy had filled a psychotropic prescription within one year prior to therapy onset, particularly antidepressants (44 %) and antipsychotics (22 %).

**Conclusions:**

The use of psychotherapy for treatment of psychiatric disorders is limited among Danish patients, although national clinical guidelines recommend it as first-line treatment of common conditions such as depressive, anxiety and obsessive-compulsive disorders.

**Disclosure:**

No significant relationships.

